# Generalized learning induced by training and tDCS is predicted by prefrontal cortical morphology

**DOI:** 10.1093/cercor/bhaf229

**Published:** 2025-08-20

**Authors:** Shane E Ehrhardt, Yohan Wards, Thomas B Shaw, Kelly G Garner, Steffen Bollmann, Jason B Mattingley, Paul E Dux, Hannah L Filmer

**Affiliations:** School of Psychology, The University of Queensland, McElwain Building, Campbell Road, St Lucia, 4072, QLD, Australia; School of Psychology, The University of Queensland, McElwain Building, Campbell Road, St Lucia, 4072, QLD, Australia; Centre for Advanced Imaging, The University of Queensland, Building 57, Research Road, St Lucia, 4072, QLD, Australia; School of Electrical Engineering and Computer Science, The University of Queensland, Building 78, Staff House Road, St Lucia, 4072, QLD, Australia; School of Psychology, The University of Queensland, McElwain Building, Campbell Road, St Lucia, 4072, QLD, Australia; School of Psychology, University of New South Wales, NSW, Australia; School of Electrical Engineering and Computer Science, The University of Queensland, Building 78, Staff House Road, St Lucia, 4072, QLD, Australia; Queensland Digital Health Centre, The University of Queensland, Health Sciences Building, RBWH, Fig Tree Crescent, Herston, 4006, QLD, Australia; School of Psychology, The University of Queensland, McElwain Building, Campbell Road, St Lucia, 4072, QLD, Australia; Queensland Brain Institute, The University of Queensland, Building 79, Research Road, St Lucia, 4072, QLD, Australia; Canadian Institute for Advanced Research (CIFAR), 661 University Ave, Toronto, ON M5G 1M1, Canada; School of Psychology, The University of Queensland, McElwain Building, Campbell Road, St Lucia, 4072, QLD, Australia; School of Psychology, The University of Queensland, McElwain Building, Campbell Road, St Lucia, 4072, QLD, Australia

**Keywords:** cognitive training, cortical thickness, decision-making, dose, tDCS, transfer, ultra-high field MRI, visual search

## Abstract

Brain stimulation shows promise as an intervention to enhance executive function, particularly when paired with cognitive training. To optimize such approaches, we must understand the potential role of individual differences in intervention outcomes. We investigated the combined effects of multi-session multitasking training and prefrontal transcranial direct current stimulation (tDCS) on generalization of performance benefits, focusing on how cortical morphology predicts performance improvements. One hundred seventy-eight individuals underwent 7 Tesla MRI before completing multisession training with online stimulation. A cognitive task battery assessed improvements in trained and untrained tasks pre- and post-training. Stimulating the left or right prefrontal cortex at 1 mA during multitasking training enhanced transfer to a visual search task. Critically, cortical morphology predicted stimulation efficacy for inducing transfer. Cortical thickness in regions beneath the stimulating anode was related to reaction time changes in the most difficult visual search condition but only for the left and right 1 mA multitasking training groups. Performance was not related to cortical thickness for the groups receiving sham stimulation, 2 mA stimulation, or 1 mA stimulation with a control training task. These results highlight the importance of individual anatomical differences in modulating tDCS efficacy and identifying specific neuroanatomical features that predict generalized performance gains from combining tDCS with cognitive training.

## Introduction

The benefits of learning are greatly enhanced when they generalize—or transfer—across stimuli, responses, and contexts. Over the past decade, considerable research has sought to determine if cognitive training paradigms can promote transferable performance benefits across various psychological operations. These include intelligence, inhibition, attention, and multitasking (Jaeggi et al. 2008; Chein and Morrison 2010; Shipstead et al. 2012; Redick et al. 2013; Simons et al. 2016). Such interventions may have applications in educational and clinical settings, given the importance of these cognitive processes (and others examined in training studies) for adaptive behavior. Generally, however, training-based interventions alone do not appear to enhance performance beyond the specific task trained (Redick et al. 2013; Melby-Lervåg et al. 2016; Sala and Gobet 2019). Thus, a key challenge is to understand the limits of learning generalization (Noack et al. 2014; Green et al. 2019; Garner and Dux 2023) and to identify the conditions required for transfer to arise (Noack et al. 2014; Garner et al. 2016; Garner and Dux 2023).

In recent years, transcranial direct current stimulation (tDCS) has emerged as a promising noninvasive brain stimulation technique for modulating cognitive performance (Filmer et al. 2013a; Filmer et al. 2013b; Dedoncker et al. 2016; Ehrhardt et al. 2022), and affording causal insights into training processes. By applying a weak electrical current to the brain via two or more electrodes placed on the scalp, tDCS can alter cortical excitability (Nitsche et al. 2003; Stagg and Nitsche 2011) and neural activity (Antal et al. 2004; Reato et al. 2010). tDCS has been shown to positively influence a number of cognitive processes including attention (Weiss and Lavidor 2012; Coffman et al. 2014), learning (Reis et al. 2009; Martin et al. 2014; Buch et al. 2017), and memory (Zaehle et al. 2011; Mancuso et al. 2016). Importantly, tDCS has also been found to induce transfer of training benefits. For example, when applied over multiple days to the left prefrontal cortex during training on single- and dual-task paradigms, visual spatial attention (assessed via a visual search task) has been shown to be enhanced, as evidenced by larger reductions in reaction times from pre- to post-training compared to control conditions (Filmer et al. 2017a; Filmer et al. 2017b; Wards et al. 2023, 2024; Ehrhardt et al. 2024).

It is widely recognized, however, that individuals’ responses to tDCS are highly variable (López-Alonso et al. 2014; Filmer et al. 2020a). This limits the potential applicability of stimulation as an intervention and hampers the optimization of protocols to modulate cognitive performance. One potential explanation for this variability is individual differences in brain morphology, which have recently been asserted to guide and constrain brain function (Suárez et al. 2020; Pang et al. 2023). Interindividual differences in cortical gray matter thickness relate to the efficacy of single-session prefrontal tDCS to modulate learning (Filmer et al. 2019a, 2020a) and decision strategies (Filmer et al. 2023). Recent modeling studies have also highlighted the influence of individual anatomical differences on the electric fields induced by tDCS (Laakso et al. 2019; Mikkonen et al. 2020). Moreover, biophysical models based on standard tissue resistances demonstrate that variations in tissue type and architectural properties like thickness significantly modify current flow and its impact on underlying neural tissue (Opitz et al. 2015; Huang et al. 2017). This relationship between cortical architecture and stimulation effects is further supported by evidence linking cortical thickness to cognitive processes (Kanai and Rees 2011), such as sustained attention (Mitko et al. 2019) and specific executive functions. Westlye et al. (2011) found that thicker anterior cingulate cortex related to better executive control, while thicker right inferior frontal gyrus correlated with reduced executive control. To wit, a thicker temporal–parietal junction cortex was associated with more efficient alerting. It is important to note that cortical thickness is not a direct measure of neuronal properties per se but results from an interaction of cellular and molecular factors, including neuronal and non-neuronal cell density, synaptic structures, and vasculature (Spalletta et al. 2018). These findings suggest a link between brain structure and distinct aspects of cognitive function.

Integrating tDCS with neuroimaging techniques allows investigation of neural factors underlying cognitive improvement from combined stimulation and training interventions (Parkin et al. 2015; Fertonani and Miniussi 2017; Polanía et al. 2018). Our previous work has shown that cortical thickness predicts single-session tDCS effects on learning (Filmer et al. 2019a). However, it remains unclear whether these brain–behavior relationships extend to multi-session stimulation protocols and transfer performance to untrained tasks.

Here, in a large-scale, preregistered study, we investigated how individual differences in cortical morphometry relate to the combined effects of multi-session multitasking training and prefrontal tDCS on the generalization of learning (previously reported in Wards et al. 2023, 2024; Ehrhardt et al. 2024). Specifically, we have shown that neurochemistry predicts tDCS transfer (Ehrhardt et al. 2024), while functional changes are related to stimulation efficacy and transfer (Wards et al. 2023, 2024). Here, we hypothesized that individual differences in cortical thickness, particularly in regions beneath the stimulating anode electrode, would predict the magnitude of the combined training and tDCS-induced effects. To preview our findings, the degree of transfer was associated with the cortical architecture proximal to the stimulating anode. These findings, in conjunction with results previously reported from the same dataset (Wards et al. 2023, 2024; Ehrhardt et al. 2024), advance our understanding of the mechanisms underlying cognitive enhancement via combined brain stimulation and training interventions.

## Methods

**Fig. 1:**
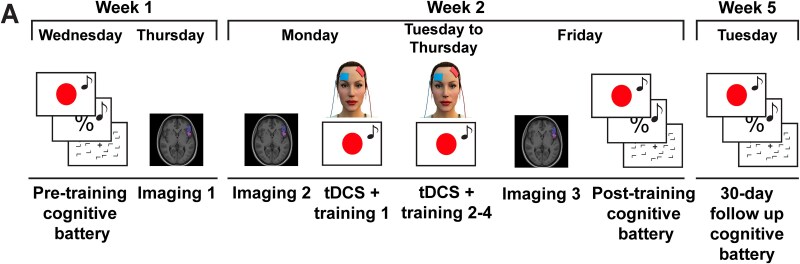
Study design and timeline. The study comprised 10 sessions over 5 weeks. In week 1, participants completed a pretraining cognitive battery on Wednesday and underwent structural MRI (imaging 1; as well as magnetic resonance imaging as reported in a previous investigation Ehrhardt et al. 2024) on Thursday. During week 2, participants underwent a second MRI session (imaging 2) on Monday (which also involved collected functional scans not used in the current study; see Wards et al. 2023, 2024), followed by four consecutive daily sessions of combined tDCS and cognitive training from Tuesday to Friday (tDCS + training 1 to 4). Participants underwent a post-training MRI and cognitive battery on Friday, and in week 5, a 30-d follow-up cognitive battery.

The study was preregistered (https://tinyurl.com/5h8u72j5), and any deviations are noted. Participants each completed 10 sessions, including pre- and postintervention test sessions, 4 training sessions that combined tDCS and training, and 3 imaging sessions (see Fig. 1). Structural scans were collected in two imaging sessions prior to training and one session after training. The pre-, post-, and follow-up intervention test sessions consisted of nine cognitive tasks that aimed to evaluate distinct cognitive processes. These tasks were conducted using Mac minicomputers (2014 model; 2.8 GHz Intel Core i5) and displayed on 24-inch ASUS VG248 monitors (144 Hz refresh rate). The tasks were run with MATLAB 2016b (The MathWorks, Inc., Natick, MA) and Psychtoolbox (Brainard 1997; Pelli 1997: http://psychtoolbox.org/). Participants were positioned approximately 57 cm away from the screen and were requested to maintain this position during task performance. The study employed a within- and between-subjects design, with participants divided into five groups. Two groups, the sham left prefrontal cortex and 1 mA left prefrontal stimulation paired with multitasking training, were double-blinded throughout the study. The other three groups served as active controls and included a dosage control group 2 mA left prefrontal combined with multitasking training), a different montage control group (1 mA to right prefrontal combined with multitasking training), and a different training task control group (1 mA applied to the left prefrontal combined with rapid serial visual presentation task training). To minimize any potential impact of task order on baseline performance measures, the order of task completion was randomized and counterbalanced for all participants and groups.

### Participants

To determine the necessary sample size for our study, a power analysis was performed using the software G^*^Power and assuming a conservative effect size of 0.3 (Cohen’s *D*) for the behavioral effects of tDCS on multitasking training, based on previous research from our lab (Garner and Dux 2015; Filmer et al. 2017a; Ehrhardt et al. 2021). The analysis showed that 33 participants per group would be needed for between-group comparisons with 95% power using an analysis of variance (ANOVA) repeated measures between factors test, and 46 participants per group for individual differences analyses using a linear multiple regression. Therefore, our goal was to recruit a maximum of 250 participants, with 50 participants in each group. However, due to the COVID-19 pandemic and associated lockdowns, the final sample size was 207 participants. The data collection period was set to end on 2021 September 28, but was extended until 2022 January 15, to reach our minimum target sample size for between-subject comparisons, without looking at the data. Out of the 207 participants, 29 were excluded for various reasons (see below), leaving a final sample of 178 participants who completed sessions 1 to 9 (as shown in Fig. 1). Of these, 167 participants completed the 30-d follow-up session 10. Table 1 provides demographic information for each group.

**Table 1:** Participant demographics, by allocated group.

	Sham	1 mA left PFC	1 mA right PFC	1 mA left PFC RSVP	2 mA left PFC
*N*	36	35	35	36	36
Age	22.56	23.09	23.26	23.22	22.19
Sex (M/F)	11/25	11/24	12/23	12/24	13/23

### Exclusions

Subjects were removed from the final sample for a variety of reasons, including incidental findings (pineal gland cysts: [6], prior to any electrical stimulation for precautionary reasons), issues with tolerating MRI scans (nausea, claustrophobia, anxiety, noisy and lengthy scanning sessions: [10]), inability to complete sessions due to COVID-related lockdowns (3), insufficient accuracy in multitasking paradigms (3), missed sessions (2), personal reasons (1), inappropriate behavior (1), discomfort with tDCS (though no adverse effects were noted): (1), taking psychoactive medication (1), and considerably reduced accuracy in a training session (1). COVID-19 lockdowns meant eight participants could not attend the 30-d follow-up session, while three did not respond to emails.

Before participating, all subjects underwent safety screenings, including a safety questionnaire, an MRI safety questionnaire, and a quiz to confirm their understanding of the techniques being used in the study, such as transcranial direct current stimulation and MRI. Exclusion criteria included a history of brain trauma, current use of psychoactive medications, and a personal or family history of epilepsy. The study was approved by The University of Queensland Human Research Ethics Committee (HREA:2009000335), and all participants provided written informed consent and were reimbursed for their time, receiving $20 AUD/h, with the total amount paid roughly ~$380 if they completed all 10 sessions. After the first session, participants were assigned to groups using an automated algorithm to balance key characteristics such as age, sex, time of day of intervention, reaction times for single and dual tasks in the multitasking paradigm, and the order in which they completed their tasks. This algorithm was designed to prevent any baseline differences between groups, like the method used in Ehrhardt et al. (2021).

### Stimulation parameters

**Fig. 2:**
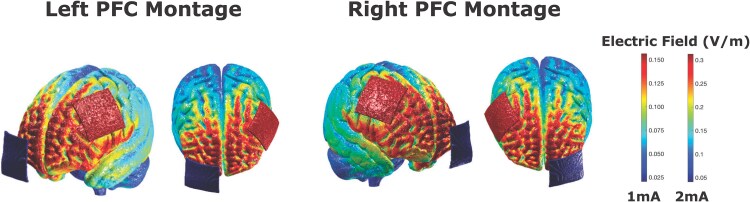
ROAST current modeling (Huang et al. 2016, 2019) for the two tDCS montages used in the study, with the anode targeting either the left or right PFC.

The stimulation was administered using a NeuroConn stimulator with two 5 × 5 cm sponges soaked in saline and equipped with rubber electrodes, held in place by rubber straps. For the group targeting the left prefrontal cortex, the anodal electrode was placed 1 cm behind F3, based on the 10-20 electroencephalogram (EEG) system, with the cathode positioned over the right supra orbitofrontal cortex, following the configuration used in several previous studies on executive function (Dedoncker et al. 2016; Filmer et al. 2017a; Filmer et al. 2017b; Ehrhardt et al. 2021). The right prefrontal cortex group had the anode placed 1 cm behind F4, with the cathode placed over the left supra orbitofrontal cortex, as seen in Fig. 2. Participants received 13 min of stimulation during training with a gradual increase and decrease of 30 s. To eliminate the influence of the time of day on tDCS outcomes, as observed in previous research (Ridding and Ziemann 2010), all sessions were conducted at roughly the same time of day, with a deviation of ±2 h. To align with the commonly used intensities of 1 and 2 mA in 95% of tDCS studies (Bikson et al. 2016), these intensities were chosen to facilitate the generalizability of the findings. Previous research has also shown that stimulation intensity has a differential effect on multitasking performance (Ehrhardt et al. 2021) and decision-making processes (Ehrhardt et al. 2022).

### Multitask training

Participants trained on a sensory–motor multitask adopted from Filmer et al. (2017a) and Ehrhardt et al. (2021), which aims to increase the speed (ie reduce information processing time), while maintaining accuracy, at which participants can execute a single or multiple decision(s). This task was also in the pre-, post-, and follow-up testing sessions for the training control-task group that trained on a rapid serial visual presentation task (as described below). Participants were presented with visual stimuli centrally consisting of a single-colored circle (either a red: RGB 237 32 36, dark green: RGB 10130 65 or dark blue: RGB 44 71151, subtending approximately 2.7° of visual angle). Auditory stimuli consisted of one of three complex tones (as used in Dux et al. 2006), presented in stereo through Shure SRH440 over-ear headphones. Each stimulus was mapped to a unique key on a keyboard (A, S, D [left hand] J, K, L [right hand]; with the participants’ index fingers on the D and J keys, respectively). The response mappings were counterbalanced across participants and groups, where half of the participants used their left hand for the auditory task and right hand for the visual task, and this was reversed for the other half of participants. Three different trial types constituted the overall multitask: single-visual (a colored circle; red, green, or blue), single-auditory (one of three complex tones), or a combined visual–auditory dual-task (one colored circle and one complex tone, presented simultaneously). In line with previous multitasking work (Dux et al. 2009), the visual and auditory stimuli were presented simultaneously (0 ms SOA) in the dual-task trials. For each trial, a centrally presented fixation square (0.4° diameter) was displayed for either 600 or 1,000 ms, followed by the stimulus presentation for 200 ms. Participants were instructed to respond by pressing the appropriate key(s) as quickly and accurately as possible. Each trial had a 2,200-ms response window starting from stimulus onset. To reduce exclusion rates due to poor performance, participants practiced at the pretraining session on both single and dual tasks until a 70% accuracy cut-off for the dual-task condition was reached. Of the three blocks of practice, the first two blocks involved 15 trials of each single task (auditory and then visual, respectively), while the third block consisted of 30 trials containing 10 trials of every type (single visual, single auditory, or dual task), in random order. Participants repeated the third block until 70% dual task accuracy was reached, and this third block was completed by participants prior to every session (pre-, post-, follow-up, & training sessions) to refresh the response mappings. Participants completed a total of 240 trials (single-visual; 80, single-auditory; 80, dual-task; 80, randomly mixed) in each session, with a 30-s break at the halfway point. Each 240-trial session lasted ~13 min.

### Control training task

To investigate the potential benefits specific to combining simulation and multitasking training, one active control group was enlisted to perform a selective attention control task—Rapid Serial Visual Presentation task (RSVP, refer to Potter and Levy 1969; Raymond et al. 1992; Dux and Marois 2009). Participants were presented with a fixation square with a diameter of 0.4°, which appears for a duration of 0.2, 0.3, 0.4, 0.5, or 0.6 s. A sequence of seven distractor numbers followed this, and participants were required to identify a single target “letter” within this numerical array (eg 4, 2, W, 5, 3, 8, 9, 7). Both numerical and alphabetical stimuli projected a visual angle of approximately 0.8°. Responses were registered through the pressing of a key on a conventional keyboard corresponding to the letter displayed (potential letters included: “A,” “B,” “C,” “D,” “E,” “F,” “G,” “H,” “J,” “K,” “M,” “N,” “P,” “R,” “S,” “T,” “W,” “Y,” and “Z”). The position of the letter was arbitrarily dispersed throughout each trial between the third and sixth positions in serial order. The experiment was divided into three blocks of 200 trials in total. The rest period between each block was dynamically adjusted to align the task duration (or training time) with the multitask. Furthermore, the presentation speed was thresholded for each participant to maintain an approximate accuracy of 70%. During the pretraining session, each stimulus was initially displayed for a duration of 100 ms. If a participant was able to accurately respond to five consecutive trials, the display duration was reduced by increments of 10 ms. However, if two consecutive trials were answered incorrectly, the display duration would be increased by 10 ms for each stimulus. The minimum duration for any given stimulus was 10 ms. Each subsequent session commenced with the final presentation duration (ie 20 ms) of the preceding pretraining session. Presentation duration, an indicator of performance (shorter durations signifying better performance), was the key dependent variable. Each 200-trial session spanned approximately 13 min. This task was also incorporated in the pre-, post-, and follow-up testing sessions, serving as a transfer task for the four groups that participated in the training multitask.

### Transfer multitask paradigm

Near transfer was assessed on a task identical in structure to the training multitask but which implemented different visual stimuli (“%,” “#,” and “&,” subtending a visual angle of approximately 1.2°) and different complex tones (Dux et al. 2006).

### Visual search

Our primary transfer task was a classic inefficient visual search task (find a “T” among distractor “L’s”), which taps spatial attention mechanisms and has previously shown transfer in two experiments combining tDCS with cognitive training (Filmer et al. 2017a; Filmer et al. 2017b). In contrast to both training tasks, this task requires a different response to different stimuli (finding a stimulus among similar distractors vs. selecting the correct response to a presented stimulus) and thus is considered a far transfer task as it primarily taxes a distinct cognitive domain. Trials first displayed a fixation dot of 0.25° diameter, which lasted for a randomly determined duration of either 0.4 or 0.6 s before disappearing. Subsequently, an array of “L” stimuli and a single target “T” stimulus appeared (total array subtended 17° of visual angle), located equidistant from one another in random positions within the search array. These two stimuli had an approximate linewidth subtending 0.2° and a height of 0.8° of visual angle. Participants were required to find the single “T” (rotated either 90° or 270°) among the distractor letter “L’s” (randomly rotated either 90° or 270°). For this task, the difficulty was manipulated by varying the number of distractor “L”s (7, 11, or 15), where participants completed 80 trials for each task difficulty, totaling 240 trials. Within a 3,000-ms time window, participants were required to use their left index finger to press the “Z” key if the “T” was rotated 270° or their right index finger to press the “M” key if the “T” was rotated 90° as quickly and as accurately as possible. Before each of the pre-, post-, and follow-up testing sessions, participants completed 15 practice trials, during which feedback was given for incorrect responses in the form of a short auditory tone. Performance was measured as reaction time to find the target, on trials with responses indicating the correct orientation of the target.

### Random-dot motion

In this task, participants viewed a random dot kinematogram displaying two sets of dots (160 black [RGB: 0 0 0] and white dots [RGB: 255255255]) moving simultaneously in either different (incongruent) or similar (congruent) directions. Each dot was 1 pixel in size, and the total circular dot display area measured 400 pixels in diameter (11°). Before starting a block of trials, participants were prompted to track dots of a specific color. They were instructed to indicate whether the target-colored dots were moving to the left or right of the vertex as accurately and quickly as possible. Task difficulty was manipulated by varying the angular offsets: [15° 75°; 15° −45°; 30° 90°; 30° −30°; −15° 45°; −15° −75°; −30° 30°; −30° −90°]. Throughout each trial, participants were required to maintain central fixation on a red dot (approximately 0.2° of visual angle). Prior to each pre-, post-, and follow-up testing session, participants completed 10 practice trials with each cued color. During the test phase, participants performed 80 trials with each cued color, with 10 trials at each of the 8 different angular offset configurations. The average accuracy and reaction time for each angular offset configuration were recorded as measures of performance.

### Go No-go with mind-wandering

Our previous research has demonstrated that tDCS can lead to increased self-reported mind-wandering during training when higher stimulation intensities (ie 2 mA) are used (Filmer et al. 2019). To assess mind-wandering in this study, participants responded to a probe appearing pseudo-randomly (every 15 to 25 trials) throughout a simple go no-go task. The probe presented a question: “To what extent have you experienced task unrelated thoughts prior to the thought probe? 1 (minimal) – 7 (maximal).” Participants had 10 s to respond, after which the go no-go task resumed. A total of 5 probes were presented per block, with 4 blocks in total. In the go no-go task, participants were familiarized with two similar yet distinct visual stimuli (a spiky and a smooth white abstract 3D shape, both approximately 1.2° in size). The “go” stimulus was pseudorandomized, with half of the participants having the spiky object and the other half having the smooth object as the “go” stimulus. The remaining object served as the “no-go” stimulus. Trials began with a fixation cross (0.5° diameter, 0.1° linewidth) displayed for a randomly determined duration of either 0.2, 0.3, 0.4, 0.5, or 0.6 s. Following this, one of the two stimuli would appear, and participants were required to respond by keypress each time the go stimulus appeared (75% of trials) while withholding their response to the no-go stimulus (25% of trials). Participants were instructed to respond as quickly as possible. The primary measure of interest for this task was the stopping accuracy on no-go trials, where participants needed to withhold their response.

### Operation span

We employed an automated version of the Operation-Span Working Memory Task (O-Span; Unsworth et al. 2005), which involves retaining information while performing a concurrent task using visual stimuli (letters and numbers). The task began with a fixation square (0.4° diameter) presented for a randomly determined duration of 0.2, 0.3, 0.4, 0.5, or 0.6 s, followed by a simple math equation [eg “(2 × 4) − 3 =? ”] displayed centrally. Participants had to click the mouse using a standard computer mouse to indicate that they had solved the problem. Subsequently, a solution to the equation (eg “5”) was presented centrally, and participants had to indicate whether the presented answer was TRUE or FALSE by clicking the mouse. After responding to the math equation, a single letter (“F,” “H,”, “J,” “K,” “L,” “N,” “P,” “Q,” “R,” “S,” “T,” or “Y,” approximately 0.8° in size) was displayed for 800 ms, which participants had to remember. Immediately following the letter presentation, another trial with the same structure would begin. At the end of each block of trials, a response screen showed all possible letters in a grid array, and participants had to click on the letters they had seen throughout the block in the correct presentation order using the mouse. The number of trials per block varied randomly (3 to 7 trials per block) to modify difficulty, with a total of 15 blocks.

Performance was assessed using a partial credit unit score, calculated by summing the fraction of correctly reported letters for each block across all blocks and dividing by the total number of blocks. This task was considered a far transfer task as it involved performing simple math operations, retaining information in the face of distraction, responding with a mouse rather than a keypress, and emphasizing accuracy over speed, clearly differentiating it from the trained multi-task.

### Digit span (forward and reverse)

#### Forward

In this task, participants were presented with a stream of digits and asked to recall them in the same order. Each correct response resulted in the addition of one more digit to recall in the next trial. Two consecutive incorrect trials led to a reduction of one digit in the subsequent trial. The task consisted of a total of 15 trials. Performance was assessed using a partial credit unit score, calculated by summing the fraction of correctly reported numbers for each trial across all trials and dividing by 15.

#### Reverse

After completing the forward component of the task as described above, participants were presented with a stream of digits and asked to recall them in reverse order. Each correct response resulted in the addition of one more digit to recall in the next trial, while two consecutive incorrect trials led to a reduction of one digit in the subsequent trial. There was also a total of 15 trials, and performance was assessed using the participant’s partial credit unit score, as in the forward component.

### Dynamic dual task

We used a continuous visuomotor tracking task combined with a perceptual discrimination task (adapted from Bender et al. 2017) to further investigate multi-tasking ability. This task combined rapid perceptual decisions (reaction time based) with a continuous tracking task (accuracy based), differentiating it from other multitask paradigms that primarily assess improvements in reaction times. The main dependent variable in the DDT is accuracy. In the tracking component of the task, participants used a computer mouse to keep a cursor (1.2°) inside a moving disk (5.5° diameter) on the screen while ignoring centrally presented shapes. The disk’s trajectory was generated by the visible target bouncing off the display boundaries or off two invisible discs in a Newtonian direction. Movements in the *x* axis and *y* axis were calculated independently, and a 1 cm protective radius was implemented around the shapes to prevent the tracking target from overlapping with them. Accuracy was recorded as the percentage of time the cursor was kept within the edges of the disk. In the discrimination component of the task, participants were cued to a specific-colored shape before the trial began. They then responded with the spacebar as quickly as possible when the target shape was presented in the center of the screen while not responding to distractor shapes and ignoring the moving disk. There were 12 possible colored shapes (red, green, blue, or yellow) represented as either a square, hexagon, or star, with an approximate size of 4.5°. Distractor shapes occurred at a 50% frequency, and shapes were presented every 2, 2.5, or 3 s during a trial. In the multi-tasking component of the task, participants performed both the tracking and discrimination components simultaneously. Multi-tasking performance was estimated by calculating the difference in accuracy between the single task trials and the dual task trials for both the tracking and discrimination components.

To ensure ~ 80% accuracy on each single-task condition, the speed of the moving target disk and the response window duration for the target shape were manipulated using a thresholding procedure before the task began in the first session. Details of this thresholding procedure can be found in Bender et al. (2017). Briefly, the tracking target speed to be used throughout the experiment was determined from 9 × 60-s tracking trials, where the disk’s speed would change (between 0.01 and 0.112 degrees per second) to maintain 80% tracking accuracy. The response window duration for the shape discrimination component was determined from 9 × 20 s trials, where the response window would change between 250 and 1,000 ms to maintain 80% discrimination accuracy. During the thresholding session, a centrally presented fixation square (0.5°) was presented. The square would turn green for 50 ms if the target shape was accurately responded to within the current response window or if a response to a nontarget shape was successfully inhibited. The square would turn red for 50 ms if the target shape was not responded to within the allotted response window or if a nontarget shape was responded to.

### MRI

**Fig. 3:**
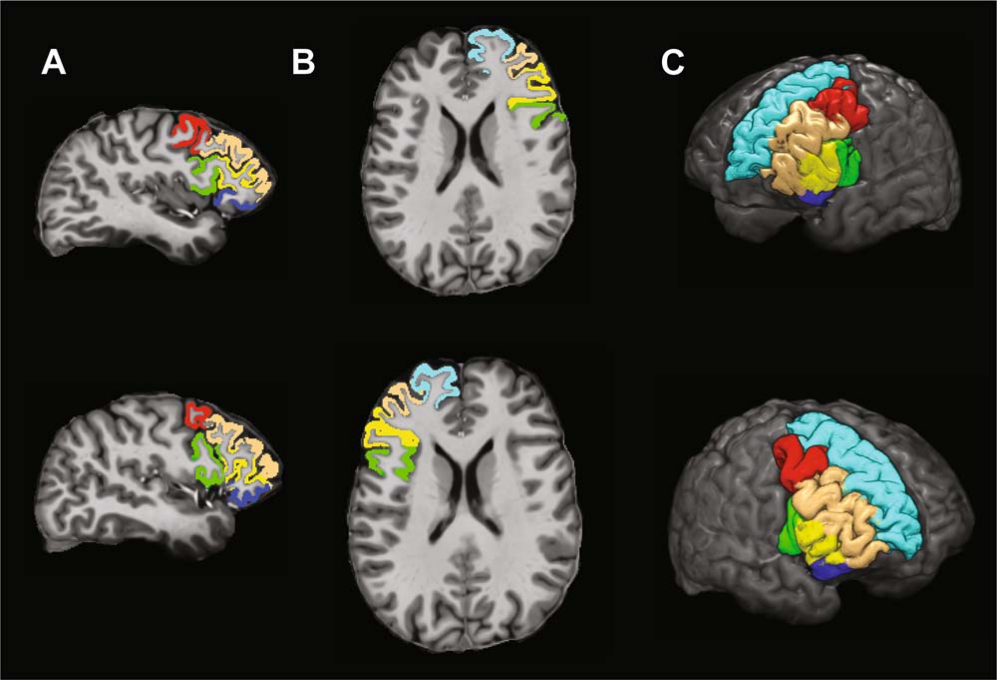
Regions of interest (ROIs) for cortical thickness analysis. A) Sagittal and B) axial views of the brain depicting the ROIs in both hemispheres. C) 3D lateral view of the brain showing all ROIs. Colors represent caudal middle frontal (red), pars opercularis (green), pars triangularis (yellow), rostral middle frontal (orange), superior frontal (light blue), and pars orbitalis (dark blue). Top row shows the left hemisphere; bottom row shows the right hemisphere. Cortical thickness measures were extracted from these ROIs using advanced normalization tools (ANTs) and the DKT-31 atlas. Median cortical thickness values across three MRI sessions were used for each participant.

Across three imaging sessions, MR images were acquired on a 7T whole-body research scanner (Siemens Healthcare, Erlangen, Germany), with maximum gradient strength of 70 mT/m and a slew rate of 200 mT/m/s and a 7 T Tx/32 channel Rx head array (Nova Medical, Wilmington, MA, USA). We acquired anatomical T1-weighted scans in each of the three separate sessions for each participant, in line with prior work demonstrating that multiple acquisitions can substantially improve segmentation consistency at ultra-high field strength (Shaw et al. 2019). We used a prototype MP2RAGE sequence (Work In Progress (WIP) 900; Marques et al. 2010; O’Brien et al. 2014) at 0.75 mm isotropic voxel size (TR/TE/TIs = 4,300 ms/3.38 ms/840 ms, 2,370 ms, TA = 6:54, GRAPPA acceleration factor = 3, and a phase partial Fourier 6/8). A total of three participants did not have usable data (likely due to movement) and were excluded from the cortical thickness analyses. Data processing was performed on Bunya: The University of Queensland Research Computing Centre. 2024. Bunya supercomputer. Brisbane, Queensland, Australia (Dell Technologies Pty Ltd 2024). T1w images were segmented using Advanced Normalization Tools (ANTs) version 2.2.3. We first constructed a population-specific template (of 50 participants) using antsMultivariateTemplateConstruction2.sh and 20 iterations to achieve good convergence. The template was then labeled using the antsCookTemplatePriors script to construct our own tissue priors for all subsequent processing. Following this atlas construction, we used the antsLongitudinalCorticalThickness.sh script with default parameters for cortical thickness and white and gray matter labeling of each subject at each time-point. Finally, we employed Joint Label Fusion with a subset of the Mindboggle-101 (Klein and Tourville 2012; Klein et al. 2017) label priors to label each participants’ DKT-31 regions (Klein and Tourville 2012) at each time-point. The ROIs included in this study were frontal regions across the two hemispheres (see Fig. 3) as we anticipated they could be influenced by stimulation targeting frontal regions. Cortical thickness estimates and volume for these regions of interest (ROIs) were calculated using ANTs including mean, min, max, and SD cortical thickness values, surface area, and volume for the given ROIs. Our analyses focused upon cortical thickness, and visual inspection of the cortical thickness images was completed. As each participant was scanned three times, we took the median cortical thickness values for each participant. The final data were checked for outliers (values > 3 SD from the mean), and none were found.

### Data analysis

The selection of independent variables (IVs) was guided by their anatomical proximity to the anodal electrode placed over the left or right prefrontal cortex. For each group and hemisphere, we fitted linear models that included brain volume as a covariable and six cortical regions: pars opercularis, pars triangularis, pars orbitalis, caudal middle frontal, rostral middle frontal, and superior frontal. All variables were *z*-scored; therefore, the beta coefficients are directly interpretable as standardized units. These regions were identified and labeled using the Mindboggle software, which employs the Desikan–Killiany–Tourville (DKT) protocol based on the Destrieux Atlas. This selection criterion for IVs ensured that the analysis focused on areas most likely to be directly influenced by the electrical stimulation, thereby improving the specificity and interpretability of the model.

Model validation and assumption checks were rigorously conducted to ensure the robustness of the linear model. Linearity and independence of the residuals were assessed using Residual vs Fitted plots. Homoscedasticity was evaluated using the Breusch–Pagan test, implemented through the ncvTest() function in R. The normality of the residuals was verified both visually, using Q-Q plots, and statistically, using the Shapiro–Wilk test. To assess multi-collinearity among the independent variables, Variance Inflation Factors (VIFs) were calculated for each, with a VIF threshold of 10 set for variable exclusion. In addition to these diagnostic procedures, the overall model significance was tested using the F-statistic. To control for Type I error due to multiple comparisons, *P*-values of the model coefficients were adjusted using the false discovery rate (FDR) correction method.

## Results

To test our hypothesis that cortical thickness under the anodal electrodes would predict the magnitude of the effects of tDCS on behavior, we extracted cortical thickness measures from six ROIs in each hemisphere (Fig. 3), selected based on their proximity to the stimulation site and their potential involvement in training outcomes (Constantinidis and Klingberg 2016; Filmer et al. 2019b). We used a combination of Bayesian and Frequentist statistics to examine our data. Linear models were constructed for each group to examine the relationship between cortical thickness and changes in reaction times from pre- to post-training or pretraining to 30-d follow-up for the training and transfer tasks.

### tDCS induces transfer to spatial attention

**Fig. 4:**
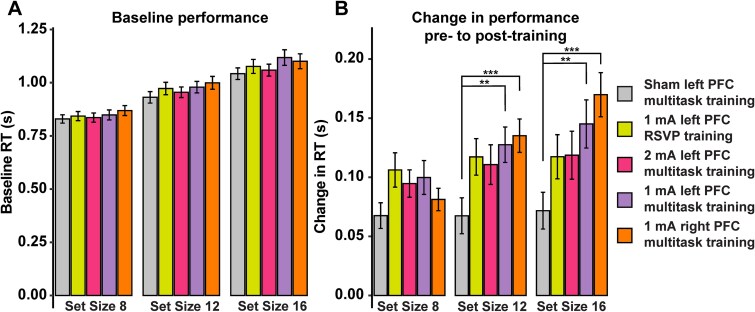
Training transfer to visual search across set sizes. A) Baseline performance across set sizes was comparable across groups. B) Change in reaction time from pre- to post-training for the transfer visual search task for distractor set sizes 8, 12, and 16. Set sizes 12 and 16 showed differences between sham and the 1 mA left and right multitasking groups. Error bars represent the standard error of the mean. ^*^ denotes BF_01_ 3 to 10; ^***^ denotes BF_01_ > 30.

As previously reported (Wards et al. 2023, 2024; Ehrhardt et al. 2024), we found substantial evidence for transfer to the visual search task in the most difficult visual search conditions (higher number of distractor items in the visual display, Fig. 4) in line with our previous work (Filmer et al. 2017a; Filmer et al. 2017b). Compared with the sham stimulation group, both the 1 mA left and 1 mA right prefrontal stimulation groups showed larger improvements in performance (faster reaction times at post- compared to pretraining) at both Set Size 12 (1 mA left, BF_10_ = 5.742; 1 mA right, BF_10_ = 14.915) and Set Size 16 (1 mA left, BF_10_ = 6.643; 1 mA right, BF_10_ = 122.788). Notably, there was strong evidence that these initial improvements were sustained when measured in the follow-up session ~30 d post-training for the highest difficulty condition of Set Size 16 (1 mA left, BF_10_ = 6.08; 1 mA right, BF_10_ = 54.284). These effects were dose-dependent; 2 mA stimulation had minimal impact on the transferable gains relative to sham (BF_10_ < 1.6 for all comparisons). Lastly, evidence of transfer arose specifically for the combination of 1 mA stimulation and multitasking training, as demonstrated by the lack of a difference in visual search performance changes after training when comparing the 1 mA RSVP training group with the sham group (BF_10_ < 1.96 for all).

### Cortical thickness predicts transfer

**Fig. 5:**
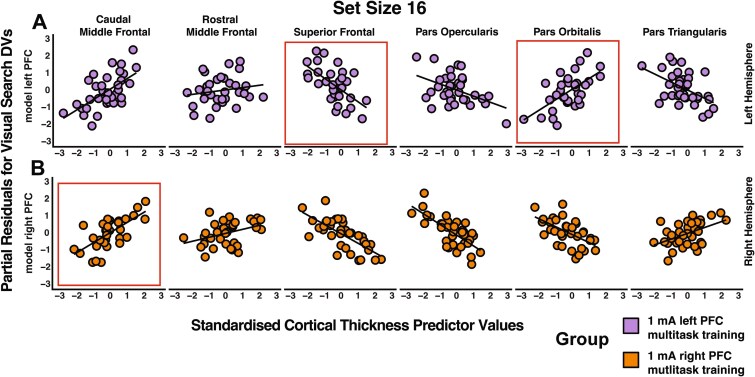
Partial residual analysis of the influence of cortical thickness predictors on changes in visual search performance (set size 16) across different electrode montages. 1 mA left- and right-prefrontal montages are represented in A & B). The *x* axis of each subplot displays standardized values of six cortical regions as predictors, while the *y* axis depicts the partial residuals. These partial residuals show the variance in reaction time changes from pre- to post-training, attributable solely to a specified predictor after adjusting for the influence of other measures. The derived linear model constructed for each group was based on the DV (visual search improvement (set size 16) from pre- to post-training), a control variable of overall “brain volume” and six IVs (cortical thickness measures from ipsilateral frontal brain regions to the anode electrode). Outline boxes indicate statistically significant associations (FDR-corrected associations only) between the cortical thickness predictor and set size 16 performance changes from pre- to post-training.

In support of our hypothesis, we observed that cortical thickness predicted the degree of transfer (reaction time improvements) from pre- to post-training, specifically for the most challenging condition (Set Size 16), in both the left and right 1 mA left prefrontal cortex stimulation groups with multitasking training (see Fig. 5 and Table 2). Crucially, only cortical regions beneath the anode electrode showed predictive relationships with transfer effects. Specifically, for the 1 mA left prefrontal group, left hemisphere regions were predictive, whereas for the 1 mA right prefrontal stimulation group, right hemisphere regions were predictive of transfer (see Table 2). Models including the contralateral regions (ie right prefrontal stimulation with left hemisphere regions and vice versa) showed no relationship between set size 16 transfer and cortical thickness (1 mA left PFC: *P* = 0.243; 1 mA right PFC: *P* = 0.371). Notably, we found no significant relationship between cortical thickness and reaction times for the less demanding conditions (Set Sizes 8 & 12; see Table 2). The observed relationships were also specific to 1 mA stimulation at either the left or right prefrontal regions combined with multitasking training. We found no significant models for the 2 mA group (*P* = 0.196, adj-*R*^2^ = 0.1077), the group that trained on the RSVP task with 1 mA left prefrontal stimulation (*P* = 0.89, adj-*R*^2^ = −0.155), or the sham stimulation paired with multitasking training (*P* = 0.583, adj-*R*^2^ = −0.04). Moreover, no individual predictors reached significance in the RSVP or sham groups (all *P*’s > 0.05 FDR-corrected).

**Table 2:** Linear regression results for the relationship between cortical thickness and improvements in visual search reaction time across tDCS conditions and set sizes, considering both ipsilateral and contralateral regions of interest. Bold values indicate significant predictors after correction for multiple comparisons using the false discovery rate (FDR) method.

Visual search regressions (ipsilateral regions with anode)					
Condition	Task	Adjusted-*R*^2^	*F*	*P*-value	Significant predictors
Sham MT	Set Size 8	0	0.92	0.507	None
	Set Size 12	0.196	2.183	0.069	None
	Set Size 16	0	0.814	0.583	None
1 mA Left PFC MT	Set Size 8	0.09	1.455	0.229	None
	Set Size 12	0.002	1.011	0.447	None
	Set Size 16	0.307	3.028	0.019	Left pars orbitalis (+), left superior frontal (−)
1 mA Right PFC MT	Set Size 8	0	0.609	0.743	None
	Set Size 12	0	0.8201	0.58	None
	Set Size 16	0.412	4.096	0.004	Right caudal middle frontal (+)
2 mA Left PFC MT	Set Size 8	0.183	2.024	0.092	None
	Set Size 12	0.079	1.359	0.267	None
	Set Size 16	0.108	1.552	0.196	None
1 mA Left PFC RSVP	Set Size 8	0.146	1.754	0.144	None
	Set Size 12	0.027	1.125	0.381	None
	Set Size 16	0	0.405	0.89	None
**Visual search control regressions (contralateral regions with anode)**					
**Condition**	**Task**	**Adjusted-*R*^2^**	***F***	***P*-value**	**Significant predictors**
1 mA Left PFC MT	Set Size 16	0.083	1.415	0.243	None
1 mA Right PFC MT	Set Size 16	0.031	1.141	0.371	None

For the 1 mA left prefrontal stimulation group, the overall model was significant [*F*(7, 25) = 3.028, *P* = 0.019, adj-*R*^2^ = 0.307], explaining 30.7% of the variance in transfer effects. Significant relationships were observed between cortical thickness and the transfer effects in two regions after FDR correction: a positive relationship with left pars orbitalis (β = 0.732 ± 0.261, *t* = 2.808, *P*-fdr = 0.033), indicating that a thicker cortex in this region was associated with greater transfer effects. Conversely, a negative relationship with left superior frontal gyrus (β = −0.650 ± 0.220, *t* = −2.956, *P* = 0.007, *P*-fdr = 0.033), suggesting that a thinner cortex in this region was associated with greater transfer effects. Additionally, the left caudal middle frontal gyrus showed a significant positive relationship (β = 0.619 ± 0.274, *t* = 2.263, *P* = 0.033), although this did not survive FDR correction (*P*-fdr = 0.076).

In contrast, for the 1 mA right prefrontal stimulation group, the overall model was also significant [*F*(7, 24) = 4.096, *P* = 0.004, adj-*R*^2^ = 0.412], explaining 41.2% of the variance. The right caudal middle frontal gyrus showed a significant positive relationship with the transfer effects (β = 0.701 ± 0.229, *t* = 3.061, *P* = 0.005, *P*-fdr = 0.038). The right pars opercularis showed a negative relationship (β = −0.455 ± 0.191, *t* = −2.387, *P* = 0.025), although this was not significant after corrections (*P*-fdr = 0.088).

### Cortical thickness models differ between groups

To further investigate the neuroanatomical basis of tDCS-induced cognitive transfer, we employed a hierarchical modeling approach to assess for differences between the groups. Specifically, we compared the 1 mA left prefrontal, and 1 mA right prefrontal stimulation groups to the sham group. Unlike the individual group analyses presented above, this approach allowed us to directly compare the effects of active stimulation to sham. Our analysis proceeded in two steps: We first fitted main effects models to establish baseline relationships between cortical thickness and transfer performance across groups. We then added group interaction terms to examine whether the relationship between cortical thickness and transfer performance differed significantly between active stimulation and sham groups.

The regression analysis comparing left prefrontal stimulation to sham showed that cortical thickness significantly moderated the effect of stimulation on transfer performance. The main effects model explained 22.9% of the transfer effect variance [*F*(8, 59) = 2.184, *P* = 0.042]. Adding thickness-by-group interactions increased the explained variance to 38.8%, an additional 15.9% (Δ*R*^2^ = 0.159, Cohen’s *f*^2^ = 0.260, *F*(14, 53) = 2.398, *P* = 0.011). We observed significant interaction effects: thicker left pars orbitalis predicted greater transfer (ie larger RT improvements) in the 1 mA left stimulation group compared to sham (β = 1.036 ± 0.391, *t* = 2.651, *P* = 0.011), while thinner left superior frontal gyrus was associated with greater transfer in the stimulation group relative to sham (β = −0.901 ± 0.385, *t* = −2.343, *P* = 0.023). These interaction effects align with the within-group relationships reported earlier, suggesting that tDCS interacts with cortical architecture in a regionally specific manner, potentially reflecting differential roles of these areas in cognitive transfer processes and their susceptibility to stimulation.

For the right prefrontal analysis, the main effects model showed a higher overall fit but smaller interaction effects when compared with sham. The main effects models explained 40.7% variance [*F*(8, 58) = 4.967, *P* = 0.0001], increasing to 49.3% with interactions (Δ*R*^2^ = 0.086, *f*^2^ = 0.170) (*F*(14, 52) = 3.61, *P* = 0.0004]. Significant interaction effects showed that thinner right pars opercularis (β = −0.627 ± 0.295, *t* = −2.128, *P* = 0.038), and thicker right caudal middle frontal gyrus (β = 0.680 ± 0.304, t = 2.239, *P* = 0.029) predicted greater transfer in the active group relative to sham. These interaction effects align with the within-group relationships reported earlier for the 1 mA right stimulation group.

Our findings suggest a nuanced relationship between cortical architecture and tDCS-induced transfer. The observed regional specificity and hemispheric asymmetries in the predictive values of cortical thickness on tDCS effects demonstrate the important role of individual neuroanatomical variation in modulating stimulation outcomes. Our results indicate that the efficacy of tDCS in closely linked to the underlying cortical architecture of the stimulated regions, aligning with prior findings (Li et al. 2015), with distinct patterns emerging for left and right 1 mA stimulation.

### Multitasking training improvements were not modulated by stimulation or cortical thickness

To investigate the specificity of tDCS-induced transfer effects, we compared performance improvements between a multitasking training paradigm and a control training task involving RSVP. Reaction times were measured to assess performance in the multitask, and stimulus presentation duration was the key measure for the RSVP task (see Methods).

As previously reported (Wards et al. 2023, 2024; Ehrhardt et al. 2024) the RSVP training group exhibited less single- and dual-task improvement relative to the other groups. Using two-sided tests, we found moderate to strong evidence for single-task improvements in the sham and both left prefrontal stimulation and multitasking groups compared with RSVP training (sham, BF_10_ = 4.588; 1 mA left prefrontal, BF_10_ = 19.123; 2 mA left prefrontal, BF_10_ = 3.558), with anecdotal evidence for the 1 mA right prefrontal and multitasking group (BF_10_ = 2.315). Dual-task improvements were also observed (sham, BF_10_ = 1739.306; 1 mA left prefrontal MT, BF_10_ = 156.638; 2 mA left prefrontal MT, BF_10_ = 376.852), and anecdotal evidence for the 1 mA right prefrontal group (BF_10_ = 2.834).

Despite reliable multitasking training effects for each multitasking training group, stimulation did not yield additional benefits compared with sham stimulation. Moderate evidence against a stimulation-induced benefit was found for both single- and dual-task components across all multitasking training groups (BF_01_ range: 1.613 to 4.049). Furthermore, there were no significant relationships between cortical thickness in any of the ROIs and changes in performance on the training tasks from pre- to post-training (all *P* > 0.05). These findings align with the lack of stimulation effects on multitasking training outcomes, consistent with previous results from our group (Filmer et al. 2017a), and suggest that individual differences in cortical thickness do not predict performance changes in this paradigm.

### Blinding was effective and there were no notable side effects

Assessing the effectiveness of blinding is crucial in noninvasive brain stimulation studies to ensure that results are not confounded by peripheral effects, such as scalp sensations (Fonteneau et al. 2019). After the post-training cognitive session, participants were asked to guess whether they received active or sham stimulation. A Bayesian ANOVA revealed no discernible differences between groups in their tendency to choose “active” stimulation (BF_10_ = 0.041). Overall, 66.8% (119/178) of participants correctly guessed their stimulation condition. However, only 14.9% (5/33) of those who guessed they received sham stimulation were correct, while 86.1% (31/36) of the sham group incorrectly guessed they had active tDCS. The sham and 1 mA left PFC group with multitasking training was double-blinded, suggesting that the sham protocol effectively blinded participants. Correct guess rates were similar across active stimulation groups (80% to 86.1%).

Participants who guessed they received active stimulation were also asked to estimate the intensity (1 or 2 mA). A second Bayesian ANOVA showed no significant differences between groups in their tendency to choose “low” intensity (BF_10_ = 0.124). Overall, 52.4% correctly guessed the intensity, not significantly different from chance. Among those who correctly guessed they were in an active condition, 66.6% accurately identified the intensity. Correct intensity guess rates were similar (74.2% to 80.8%) for 1 mA groups but lower (37.9%) for the 2 mA group. These results indicate that stimulation location, intensity, and training task did not significantly influence participants’ ability to discern their stimulation condition. The sham protocol was effective, with most sham participants incorrectly guessing they received active stimulation. Overall, the results suggest that blinding was effective and stimulation efficacy was likely not compromised by participants’ awareness of their condition.

Participants did not consistently report discomfort attributable to the stimulation. We collected ratings between 1 and 5 for sensations related to headache, neck, scalp, tingling, itching, sleepiness, concentration, mood, and “other” before and after each stimulation session. Contrasting the changes in each of these sensations between the stimulation and sham groups showed no statistically significant differences in any sensation, except for the “itching” sensation for the RSVP training group vs Sham (*P* = 0.039, Bonferroni corrected). Considering this group had an identical dose and montage to another group that did not differ to sham for any reported sensation (1 mA left PFC anodal stimulation), and the dose was the same as the 1 mA right PFC anodal group that also did not differ to sham, we concluded that this stimulation montage doesn’t consistently produce negative sensations. Furthermore, no other side effects were reported except for one participant who reported “some perceptive changes” to their vision, along with minor changes to their sleepiness and concentration.

### Transfer effects were domain-specific

We investigated whether the observed transfer effects were present in other cognitive domains, using a set of tasks selected to assess a broad range of cognitive functions that allowed us to determine the specificity of the transfer effects. The battery included assessing working memory (forward and reverse span, operational span), response inhibition (go/no-go), information processing speed (RSVP), multitasking (single/dual task with varying stimulus onset asynchrony), dynamic multitasking (shape discrimination with visuomotor tracking), and selective attention (random dot motion). We found limited evidence for the influence of stimulation and/or training on performance across this wide range of tasks (BF_10_ = 0.244 to 1.250 for all conditions). Furthermore, there were no baseline differences between any groups for any of the tasks (for all conditions, BF_10_ < 3). These findings suggest that, while multitasking training and 1 mA prefrontal cortex stimulation (left or right) facilitated far transfer to spatial attention, the scope of this transfer is narrow, helping to establish the parameter space for the observed transfer effects.

## Discussion

Cortical thickness in specific prefrontal regions predicts the degree of cognitive transfer induced by tDCS but only under specific stimulation parameters. Using ultra-high field (7T) MRI and a rigorous, multi-session experimental design with 178 participants, we found that pairing 1 mA tDCS targeting either left or right prefrontal regions combined with multitasking training can induce transfer to an untrained visual search task, particularly for the most difficult conditions. With the growing recognition of individual differences relating to variability in brain stimulation studies (Krause and Cohen 2014; Luber et al. 2017; Filmer et al. 2020b), we investigated the influence of cortical gray matter thickness in regions proximal to the anode electrode. For the 1 mA left prefrontal stimulation, thicker cortex in the left pars orbitalis and thinner cortex in the left superior frontal gyrus predicted greater transfer. Conversely, for 1 mA right prefrontal stimulation, thicker cortex in the right caudal middle frontal gyrus and thinner cortex in the right pars opercularis were associated with enhanced transfer. Importantly, these relationships were observed only under low-intensity (1 mA) stimulation conditions (not 2 mA) and were specific to transfer from multitasking training to an untrained visual search task.

Our findings advance understanding by demonstrating how specific neuroanatomical features relate to tDCS efficacy, aligning with recent work on the impact of individual brain anatomy on electric field distribution (Laakso et al. 2019; Mikkonen et al. 2020). The observed regional specificity and absence of relationships in sham conditions or with the trained task suggest that these structure–function associations are not due to nonspecific stimulation effects or broad correlations between brain morphology and cognitive abilities. Rather, they would seem to reflect the selective engagement of specific neural circuits by tDCS. This interpretation aligns with models of transcranial electrical stimulation, including tDCS, that demonstrate how seemingly simple electrical inputs can orchestrate complex changes in brain activity (Krause et al. 2023). tDCS may interact with intrinsic neural circuits in a manner dependent on local cortical architecture, amplifying latent capacity for generalizable improvements. Furthermore, recent perspectives on state-dependent brain stimulation effects (Li et al. 2019; Bradley et al. 2022; Hartwigsen and Silvanto 2022) suggest that tDCS appears to selectively modulate existing neural processes, revealing structure–function relationships that are typically subthreshold under normal conditions.

Our behavioral results replicate previous findings from our lab. In line with others, we found the effects were dose-dependent (Nikolin et al. 2018; Paracampo et al. 2018; Filmer et al. 2019;Weller et al. 2020; Ehrhardt et al. 2021, 2022) and specific to the stimulation parameters and training task. Importantly, the transfer effects were specific to the combination of tDCS and multitasking training. Neither tDCS combined with RSVP training, nor multitasking training alone (sham tDCS), were sufficient to induce transfer to the visual search task. The dose-dependent nature of the transfer effects, with 2 mA stimulation not showing transfer effects (relative to sham), or correlations with cortical thickness, highlights the importance of optimizing stimulation parameters. While increasing stimulation intensity has been proposed as a means of reducing individual variability (Esmaeilpour et al. 2018), our findings suggest that this approach may not be effective when stimulating finely tuned prefrontal regions. Specifically, increases in stimulation intensity (ie 2 mA) may minimize or negate performance improvements in certain paradigms (Weller et al. 2020; Ehrhardt et al. 2021, 2022) potentially due to the nonlinear—possibly inverted U-shaped—relationship between tDCS dose and behavioral outcomes. This may reflect a balance between Hebbian and homeostatic plasticity (Turrigiano and Nelson 2000), whereby at higher intensities, homeostatic mechanisms may act to dampen stimulation induced cortical excitability (Batsikadze et al. 2013), potentially reversing or nullifying behavioral gains (Hoy et al. 2013; Ehrhardt et al. 2021; Wards et al. 2023). These findings suggest that higher-intensity stimulation does not linearly enhance task performance and may instead overshoot an optimal window of influence. Moreover, the functional role and neurochemical sensitivity of the prefrontal cortex may make it particularly susceptible to these nonlinearities. While higher stimulation intensities such as 4 mA have been shown to enhance learning in the primary motor cortex (Hsu et al. 2023; Leow et al. 2024), this region is cytoarchitecturally and functionally distinct from prefrontal areas (Pinto and Dan 2015; Bakken et al. 2021). It remains unclear whether such intensities would be beneficial for prefrontal stimulation in cognitive paradigms. Taken together, our results highlight the need for fine-grained, region-specific optimization of stimulation intensity and suggest that 1 mA may provide a favorable balance between efficacy and tolerability for modulating prefrontal circuits during cognitive training.

Our findings demonstrate regional variability in the associations between cortical thickness and transfer effects, with thicker cortex in the left pars orbitalis and right caudal middle frontal gyrus, and thinner cortex in the left superior frontal gyrus and right pars opercularis, being associated with greater transfer in the respective stimulation conditions. This regional specificity suggests that the influence of cortical thickness on tDCS outcomes is not uniform across the cortical regions we investigated but rather depends on the location and underlying neural architecture of the broader stimulated area. The left hemisphere regions (pars orbitalis and superior frontal gyrus) that showed significant associations with transfer effects are anatomically distant from each other, while the right hemisphere regions (caudal middle frontal gyrus and pars opercularis) are closer to each other and to the targeted stimulation site. This anatomical proximity may have implications for the spread and intensity of the induced electric field, as well as the functional interactions between the stimulated regions (Bikson and Rahman 2013; Opitz et al. 2015). The differential involvement of prefrontal cortical areas between left and right stimulation effects suggests that tDCS may modulate distinct neural processes lateralized to each hemisphere. For instance, left prefrontal stimulation might preferentially enhance processing speed (Dux et al. 2006), while right prefrontal stimulation could improve spatial attention (Shulman et al. 2010), both ultimately contributing to similar gains in visual search performance. This view aligns with established hemispheric asymmetries in prefrontal cortex function (Vallesi 2012; Cai et al. 2013).

Differences in cortical thickness and cortical folding patterns across regions may affect current flow and distribution (Miranda et al. 2013; Opitz et al. 2015), due to differences in resistivity between neural tissues (Alekseichuk et al. 2019) with thicker or thinner cortical gray matter affecting current channeling and regional stimulation effects. These differences may also alter the balance of excitation and inhibition (Krause et al. 2013), modulate the efficiency of information processing (Westlye et al. 2011), and ultimately shape the behavioral response to stimulation. Furthermore, cortical thickness itself may relate to task performance (Bi et al. 2014) and individual susceptibility to stimulation-induced modulations. While the exact mechanisms remain uncertain, our findings suggest that individual differences in brain structure play a crucial role in modulating tDCS effects on cognition, aligning with previous work highlighting the involvement of prefrontal regions in decision-making (Filmer et al. 2019a). This highlights the need for individually tailored stimulation protocols that account for factors such as cortical morphology and neurochemistry (Filmer et al. 2020a; Filmer et al. 2023).

Although we focused here on cortical thickness as a morphological predictor of stimulation efficacy, a complementary approach involves modeling the predicted distribution of induced current using subject-specific electric field simulations. This technique can provide valuable insight into the spatial targeting and variability of stimulation effects across individuals in relation to cortical anatomy (Bhattacharjee et al. 2022). However, in previous work (Filmer et al. 2019a), we found that including current intensity estimates from group averaged modeling did not improve the predictive performance of models already accounting for cortical thickness. This suggests that, at least in prefrontal regions and with the specific montages used in this study, thickness may already serve as a proxy for key features influencing current distribution. Nevertheless, individualized current modeling remains an open and promising area for investigation. A future direction for our group is to combine individualized current modeling with neurite orientation and density metrics derived from NODDI, enabling a more biophysically informed account of how structural microarchitecture shapes electric field propagation and stimulation outcomes. This multimodal approach may help clarify when and where current modeling adds predictive value beyond anatomical morphology alone and ultimately inform the development of precision-targeted stimulation protocols.

Several limitations should be acknowledged when interpreting the present findings. First, while we identified associations between cortical thickness and stimulation-induced behavioral transfer, our models do not capture all potential sources of variability in tDCS efficacy. Many factors beyond cortical morphology likely contribute to individual differences in stimulation response. These include experimental variables such as the consistency of electrode placement across sessions; participant-related factors such as fatigue, alertness, and motivation; and neurobiological influences such as underlying neurochemical concentrations (Filmer et al. 2019b) and white matter microstructural profiles (Shahid et al. 2013). The role of such variables was not directly modeled here, and their influence remains an open question. Second, our analysis was limited to a specific set of anatomically defined cortical regions selected for their proximity to the stimulating electrode. However, stimulation effects are not necessarily constrained to the immediate vicinity of the anode (Weber et al. 2014). Due to the distributed nature of electrical current flow and the brain’s intrinsic connectivity, regions outside our preselected ROIs—including distal, functionally connected areas—may have also been modulated by tDCS and contributed to behavioral outcomes. Thus, while our focus on local cortical thickness improves anatomical specificity, it does not preclude the involvement of broader network-level processes. Finally, although we observed robust structure–behavior relationships, the mechanisms underlying these associations remain uncertain. Cortical thickness may influence the local distribution of electric fields, altering the amplitude or orientation of current flow within cortical circuits. Alternatively, thickness may index a combination of other neurobiological properties, such as cytoarchitectural composition and neurite density, that, in turn, modulate susceptibility to stimulation. Given the correlational nature of our findings, we cannot disentangle whether the observed relationships reflect direct modulation by current flow, indirect network-level interactions, or other unmeasured factors. Future work incorporating concurrent neuroimaging, electric field modeling, and broader anatomical metrics will be critical for elucidating the precise mechanisms linking cortical morphology to tDCS-induced behavioral change.

In conclusion, the current results reveal that the combination of prefrontal tDCS and multitasking training can induce transfer to an untrained visual search task, with the degree of transfer being related to individual differences in cortical thickness in regions under the anodal electrode. These findings, in conjunction with the results reported by Wards et al. (2023, 2024) and Ehrhardt et al. (2024), advance our understanding of the mechanisms underlying cognitive enhancement via combined brain stimulation and training. By considering individual differences in brain structure and function, future research can work toward developing personalized stimulation protocols that optimize the efficacy of cognitive interventions.
